# A comparative study between microwaves and ultrasound assisted in- situ reduction of platinum supported γ-Al_2_O_3_ using different organic templates for enhanced catalytic activity and potential applications

**DOI:** 10.1038/s41598-026-52286-0

**Published:** 2026-05-20

**Authors:** Rasha S. Mohamed, Heba M. Gobara, Fikry H. Khalil, Salah A. Hassan

**Affiliations:** 1 Catalysis Department, Egyptian Petroleum Research Institute, P.O. Box 11727, Nasr City, Cairo, Egypt; 2 Chemistry Department, Faculty of Science, Ain Shams University, Abbassia, Cairo, 11566 Egypt

**Keywords:** Mesoporous alumina, Microwave assisted solution, Ultrasonic approach, N-hexane dehydrocyclization, Ethanol dehydration, Cyclohexane dehydrogenation., Chemistry, Materials science, Nanoscience and technology

## Abstract

In this study, mesoporous alumina was synthesized and modified with two different surfactants: cetyltrimethylammonium bromide (CTAB), a cationic surfactant, and pluronic P123, a non-ionic surfactant, using the sol-gel method. The synthesis involved the co-assembly of aluminum precursors with the surfactants, followed by calcination to remove the templates, resulting in a porous alumina structure that serves as a support. Platinum (Pt) was then loaded onto the surfactant-modified alumina support (CTAB) at a concentration of 0.9 wt% Pt using microwave-assisted solution (MAS) and ultrasonic techniques. The synthesized catalysts were characterized using nitrogen adsorption-desorption, X-ray diffraction (XRD), thermal analysis, and transmission electron microscopy (TEM) to assess their textural properties, thermal stability, and morphology. The catalytic activity of the Pt-loaded alumina catalysts was evaluated for n-hexane dehydrocyclization, cyclohexane dehydrogenation, and ethanol dehydration. Both the ultrasonic technique and microwave irradiation were employed as in situ reduction methods to produce stable, well-dispersed platinum nanoparticles with average diameters not exceeding 6 nm. The catalytic performance results indicated that the 0.9 wt% Pt/Al₂O₃ (MAS) nanocatalyst outperformed its US-prepared counterpart. It achieved the highest conversion rate of 86% in cyclohexane dehydrogenation at 450 °C. In n-hexane dehydrocyclization, the MAS catalyst produced a maximum benzene yield of 48%. Additionally, in ethanol dehydration, it demonstrated superior activity with a maximum ethylene yield of 52% under the tested conditions.

## Introduction

Mesoporous nanosized alumina is recognized as a key material due to its wide-ranging potential in various applications, including catalysis, photocatalysis, optical materials, and electronic ceramics^1–3^. Its unique physicochemical properties—such as a high elastic modulus, excellent thermal and chemical stability, large specific surface area, and distinctive optical characteristics—make it particularly appealing for advanced functional application^4–7^. Alumina exists in several metastable polymorphs, including γ, η, δ, and θ phases, alongside the thermodynamically stable α-alumina phase. Among these, γ-alumina is crucial as an intermediate phase in phase transformations and is widely used as a nanomaterial due to its high surface area and strong interactions with active catalytic species^8,9^. Alumina powder can be synthesized using various methods such as vapor condensation^10^, vapor decomposition^11^, combustion synthesis^12^, thermochemical synthesis^13^, sol-gel processing^14,15^, chemical precipitation^16,17^, sputtering^18^, and thermal decomposition^19^. These techniques are essential for controlling the physicochemical properties of alumina, which enhances its use as a functional nanomaterial^20–24^.Among these methods, a common strategy involves templating surfactant micelle structures or using chemical additives like glucose and hydroxycarboxylic acids as structure-directing agents. These approaches not only allow for better control over particle morphology and pore structure but also promote the development of highly dispersed and stable catalytic systems^25–27^. Several synthesis routes have been reported for preparing mesoporous γ-Al_2_O_3_ with tailored structural and textural properties, allowing for its application in various catalytic processes. Liu et al. (2010)^28^ synthesized macro–mesoporous γ-Al₂O₃ by dissolving citric acid in ethanol, followed by the addition of P123. After four days of aging, a transparent solid was obtained, which, when calcined at 400 °C, yielded a material with bimodal pore sizes of approximately 30 and 70 nm. Mokaizh (2022)^29^ demonstrated that pH significantly influences the formation of γ-Al₂O₃ nanostructures; boehmite (AlO(OH)) was produced at pH 8, while bayerite formed at pH values above 9, decomposing into mesoporous γ-Al₂O₃ upon calcination at 550 °C. The resulting material exhibited a specific surface area of 261 m² g⁻¹ and an average pore diameter of 4.86 nm. Similarly, Gholizadeh et al. (2023)^30^ prepared γ-Al₂O₃ with high surface area and pore volume by precipitating sodium aluminate with oxalic acid in an aqueous solution. Crystalline γ-Al₂O₃ was obtained at pH 8–9 and 93–96 °C, followed by calcination above 400 °C, resulting in well-defined mesoporous structures suitable for catalytic applications^31^. More recently, Maleeka (2022)^32^ employed a sol–gel approach using aluminium isopropoxide and P123 in ethanol, with HCl and phthalic acid as additives. This method involved a two-step calcination at 400 °C and 800 °C, producing mesoporous γ-Al₂O₃ with a specific surface area of 226.37 m² g⁻¹ and a pore volume of 0.31 cm³ g⁻¹. Collectively, these studies highlight the significant influence of synthesis strategies- including templating, pH adjustment, precipitation, and sol–gel processing on the textural properties and catalytic potential of mesoporous γ-Al₂O₃.

Conventional preparation methods for supported metal catalysts generally involve ion-exchange^33^ or impregnation techniques^34^, where the support surface is treated with aqueous or alcoholic solutions of metal salts, followed by drying, calcination, and subsequent reduction in a hydrogen atmosphere to produce the active metallic phase^35,36^. Despite their simplicity and scalability, these approaches often suffer from limitations such as the agglomeration of metal species during thermal treatment, leading to uneven metal dispersion and a broad particle size distribution, which can adversely affect catalytic performance^37,38^. Consequently, considerable research efforts have focused on developing advanced synthesis routes capable of controlling particle size and dispersion at the nanoscale^39^. Alternative strategies, including electrochemical reduction, vapor deposition, microwave-assisted synthesis, and sonochemical techniques, have been explored to achieve uniform metal distribution and enhanced catalytic activity^40–42^.

In recent years, advanced synthesis techniques like microwave-assisted and sonochemical methods have gained significant attention for creating heterogeneous metal colloid catalysts^43,44^. These methods provide clear advantages over traditional thermal processes by facilitating rapid and uniform heating, which encourages homogeneous nucleation and reduces particle agglomeration^45^. Notably, microwave-assisted synthesis has become a particularly promising approach due to its high energy efficiency and short reaction times^46^. This efficiency arises from the direct interaction between microwave radiation and the reaction medium, which minimizes dielectric losses and enhances catalyst dispersion^47,48^.

For effective synthesis under microwave irradiation, the reaction medium must effectively absorb electromagnetic energy and convert it into heat^49^. This efficiency is primarily determined by the material’s dielectric loss factor, commonly referred to as the loss tangent^50^. Ethylene glycol, which is often used as a solvent in microwave-assisted synthesis, has a relatively high loss tangent of 1.35 at 2.45 GHz, allowing for rapid and uniform heating^51,52^. Furthermore, as noted by Chavan et al. (2022)^53^, ethylene glycol not only creates an effective thermal environment but also acts as a mild reducing agent, promoting the in situ formation of metal nanoparticles. In parallel, the application of ultrasonic (US) treatment provides complementary benefits by improving mass transport, reducing the thickness of the diffusion layer^54^, and altering the surface morphology of the reacting materials. The strong cavitation effects produced by ultrasonic waves increase the available surface area for reactions and encourage uniform particle nucleation^55^. As a result, metal particles can be deposited and reduced simultaneously without requiring an external heating stage, marking a significant advancement over traditional synthesis methods^56,57^.

Platinum-supported alumina (Pt/Al₂O₃) nanocatalysts are among the most widely utilized catalysts in petrochemical and petroleum refining industries due to their superior catalytic performance, thermal stability, and resistance to sintering^58,59^. These catalysts are fundamental in key industrial processes such as catalytic reforming^60,61^, hydrocracking^62^, hydrogenation^58^, and isomerization^63^, where they facilitate the conversion of heavy hydrocarbons into lighter, more valuable products and improve fuel quality through enhanced octane numbers^64,65^.

Global demand for petroleum-based fuels and chemical intermediates is increasing annually due to population growth, industrial expansion, and increasing transportation needs^66,67^. As a result, developing efficient and durable Pt/Al₂O₃ catalysts has become a crucial area of research focused on optimizing resource utilization and reducing operational costs^68,69^. Recent advancements in nanotechnology have enabled precise control over metal dispersion, particle size, and metal–support interactions, all of which significantly affect catalytic activity and selectivity^70^. Therefore, understanding and managing these parameters is essential for creating high-performance catalysts that satisfy the stringent requirements of today’s energy and chemical industries^71^.

Ethylene is a crucial feedstock in the petrochemical industry, primarily used as a precursor for producing a variety of chemicals, especially polyvinyl chloride (PVC)^72,73^. In addition to its traditional uses, ethylene can be transformed into isoparaffins and aromatic hydrocarbons through catalytic processes such as isomerization, hydrogenation, dehydrogenation, and dehydrocyclization^74^. These conversion methods are vital for enhancing the physicochemical and technical properties of petroleum-derived fuels, including gasoline, jet fuel, and diesel oil, ultimately improving combustion efficiency, stability, and overall fuel performance^75,76^.

This study focuses on the synthesis of mesoporous γ-Al₂O₃ using different surfactants (CTAB and P123, to optimize the pore structure. It also investigates how different preparation conditions affect the dispersion and structural properties of a 0.9 wt% Pt/Al₂O₃ nanocatalyst. The catalyst was produced through two methods: ultrasonic-assisted (US) synthesis and microwave-assisted solution (MAS) synthesis, both of which included an in-situ reduction step to promote uniform metal distribution and improve metal–support interactions. The main aim of this research was to identify the relationship between the physicochemical properties of the synthesized materials and their catalytic performance in converting model hydrocarbons specifically n-hexane, ethanol, and cyclohexane. This systematic comparison offers new insights into how the choice of synthesis pathways and surfactants affects active metal dispersion, ultimately impacting catalytic efficiency and selectivity.

## Experimental

### Preparation of mesoporous alumina nanoparticles

All the chemicals used were analytical grade and 99.9% pure. γ- alumina support (Al_2_O_3_) nanopowder was prepared by sol–gel method using aluminum nitrate nonahydrate (Al (NO_3_)_3_·9H_2_O) (El-Nasr Chemicals) as a precursor. 0.125 M solutions of ammonium bicarbonate and aluminum nitrate were simultaneously added from separate burettes into 400 mL of vigorously stirred deionized water. The addition was performed dropwise under continuous stirring to ensure homogeneous mixing. The solution volumes were adjusted according to the stoichiometric requirements of the precipitation reaction.

Approximately 36.8 g of aluminum nitrate (Al(NO_3_)_3_·9H_2_O), equivalent to 0.098 mol, was utilized to prepare about 5 g of γ-Al_2_O_3_. The gel formation reaction occurred at a temperature of 70 °C for 3 h, with the pH maintained between 7.5 and 8.5 using dilute solutions of nitric acid or sodium hydroxide. After the reaction, the precipitate was aged, filtered, thoroughly washed with deionized water, and dried. To investigate the impact of organic templates on the structural properties of the alumina support, various surfactants were incorporated during the synthesis of alumina nanopowders. Cetyltrimethylammonium bromide (CTAB) was added in two different amounts (1.0 g and 2.5 g), while 2.5 g of Pluronic P123 (PEG-PPG-PEG) was used as a non-ionic template^77^. The precipitate was filtered and washed with warm deionized water, followed by ethanol and then acetone to prevent contamination from sodium ions. It was then allowed to air dry at ambient temperature. The dried precipitates were calcined in a programmed furnace at 550^°^C for 5 h in air, with a heating ramp of 2^°^C per minute, to produce Al_2_O_3_ nanopowders^78^. The as-prepared alumina nanopowders utilized as supports were denoted as; A (parent alumina), AC_1_ (1.0 g CTAB), AC_2.5_ (2.5 g CTAB), and AP (2.5 g P123). To enhance clarity, the synthesis conditions and variations of the samples are summarized in Table 1.

**Table 1:** Preparation Conditions for Mesoporous γ-Al₂O₃ Supports.

Mesoporous γ-Al_2_O_3_	Template Type	Template Amount (g)
**A**	None	—
**AC**_**1**_	CTAB	1.0
**AC**_**2.5**_	CTAB	2.5
**AP**	Pluronic P123	2.5

### Preparation of nanocatalysts

**Fig. 1:**
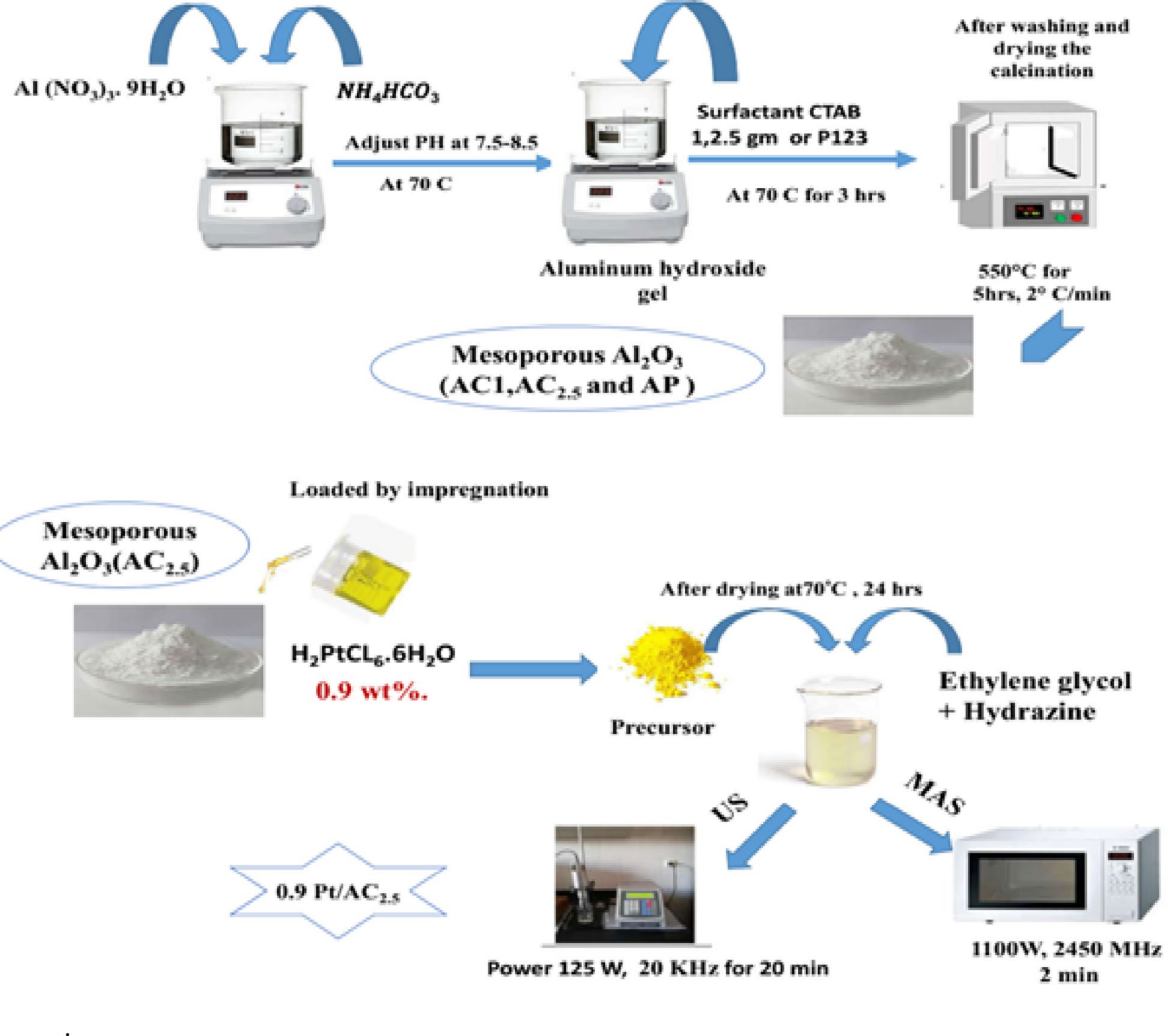
Schematic diagrams of mesoporous alumina by different method and 0.9 wt% Pt/AC_2.5_ catalysts.

The synthesis procedure for mesoporous γ-Al_2_O_3_ and Pt-loaded catalysts is illustrated in Fig. 1. For catalyst preparation, the mesoporous alumina support (AC_2.5_) was impregnated with an aqueous solution of hexachloroplatinic acid (H₂PtCl_6_·6 H₂O) to achieve a platinum loading of 0.9 wt%. The impregnated sample was then dried at 70 °C for 24 h. The reduction of the Pt precursor was conducted using two methods: (i) ultrasonic-assisted reduction (US), where 50 ml of ethylene glycol (EG) and a few drops of NaOH were added to the impregnated Pt precursor solution, followed by the dropwise addition of 5 ml of hydrazine monohydrate. The mixture was then placed in an ultrasonic apparatus (VCX-750, Sonic and Materials, Inc.), equipped with a titanium probe (13 mm in diameter) that operated at a fixed frequency of 20 kHz and variable electric output power up to 125 W for 20 min. The solid products were washed multiple times with deionized water and acetone, then rinsed with absolute ethanol to eliminate any residual impurities. Finally, the samples were dried in a vacuum oven at 70 °C for 24 h^79^. (ii) The reduction procedure previously described for the ultrasonic method was modified by using microwave irradiation instead of sonication. The resulting mixture was subjected to microwave irradiation in a Kenwood microwave oven (1100 W, 2450 MHz) at repeated intervals of 2 min^80^. After irradiation, the solid products were washed several times with deionized water and acetone, and then rinsed with absolute ethanol to eliminate any residual impurities. Finally, the catalysts obtained were dried in a vacuum oven at 70 °C for 24 h. Figure 1 illustrates the overall synthesis process for mesoporous alumina and the Pt/AC_2.5_ catalysts. The Pt-loaded catalyst supported on AC_2.5_ and reduced using the ultrasonic method is referred to as 0.9 wt% Pt/AC_2.5 (US)_, while the catalyst prepared via the microwave-assisted method is referred to as 0.9 wt% Pt/AC_2.5 (MAS)_^61,80^.

### Characterization

The textural properties of the synthesized nano-alumina and modified nanocatalysts were characterized through N₂ adsorption–desorption isotherms, measured at − 196 °C using a NOVA 3200 apparatus (Quantachrome, USA). Before analysis, all samples were degassed under vacuum (10⁻⁴ Torr) at 300 °C overnight. The specific surface areas (SBET) were determined from the adsorption isotherms using the multipoint BET equation within the relative pressure (P/P_₀_) range of 0.05–0.3. The pore size distribution (PSD) was calculated using the Barrett–Joyner–Halenda (BJH) method based on the desorption branch of the isotherms.

Powder X-ray diffraction (XRD) patterns were obtained using a Bruker D8 Advance diffractometer with Cu Kα radiation (λ = 1.5418 Å)^81^. Thermogravimetric and differential scanning calorimetric (TGA–DSC) analyses were performed on a simultaneous SDTQ 600 instrument (TA Instruments, USA) under a nitrogen atmosphere at a heating rate of 10^°^C min^⁻¹^. The morphological features of the samples were examined using high-resolution transmission electron microscopy (HRTEM) with a JEOL JEM-2100 instrument (Japan), which offers a spatial resolution of 0.143 nm and a magnification of up to 1.5 million time. Metal dispersion, average platinum crystallite size, and metallic surface area were determined using static H_2_-chemisorption with a Micromeritics ASAP 2020 analyzer. Prior to measurements, samples were reduced in situ at 450 °C for 2 h under H_2_ flow. They were then evacuated for 1 h to eliminate adsorbed species before recording adsorption isotherms at 25 °C^82^.

### Catalytic activity measurement

The catalytic performance of the supported nanocatalyst (Pt/AC_2.5_) was assessed by measuring the conversion of cyclohexane, n-hexane and ethanol, using a microcatalytic pulse reactor. The reactor effluent was passed through a chromatographic column for product separation and identification, utilizing a flame ionization detector (FID) connected to a computerized data acquisition system^83^. Product identification was carried out by comparing the retention times of the detected peaks with those of standard reference compounds and literature data under identical operating conditions. For product analysis, a stainless-steel column measuring 200 cm in length and 0.3 cm in internal diameter was packed with acid-washed PW Chromosorb (60–80 mesh) impregnated with 15 wt% squalane. Reactions were conducted at atmospheric pressure over a temperature range of 250–450 °C, with a constant hydrogen flow rate of 50 mL min⁻¹. A fixed amount of catalyst was used in all experiments. Catalytic measurements employed a microcatalytic pulse technique, where successive pulses of reactants were injected over the catalyst bed. The effective contact time was determined by the carrier gas (H₂) flow rate and the pulse injection conditions. All experiments were performed under identical conditions to ensure reproducibility. Before each catalytic test, the catalysts were reduced in a hydrogen stream at 450 °C for 2 h to completely convert platinum species to their metallic state (Pt⁰) and to achieve a fully activated and uniform surface^84^. This procedure is standard in heterogeneous catalysis and does not interfere with the primary objective of this study, which is to assess how different synthesis and reduction methods (ultrasonic-assisted and microwave-assisted synthesis) affect the physicochemical properties of the catalysts. By ensuring that all catalysts are tested under identical and comparable reaction conditions, any differences in catalytic performance can be attributed to the preparation and reduction methods, rather than to variations in the oxidation state of platinum^85^. A few preliminary pulses of the reactants were introduced to establish steady-state conditions before recording data. During the analysis, the chromatographic column temperature was held at 50 °C. Catalytic activity for ethanol and cyclohexane conversions was expressed in terms of yield, total conversion, and product selectivity, calculated using the following equations^86^:

1 $${\rm Ethanol\: or\: cyclohexane \:conversion \:(wt \%)\: is \:calculated \:as \:the \:sum \:of \:the \:yield \:percentages \:of \:each \:component \:in \:the \:products }$$

2 $${\rm The \:component \:yield \:(wt \%) \:is \:determined \:by\: dividing \:the \:quantity \:of \:the \:component \:by \:the \:total \:quantity \:of \:all \:produced \:components }$$

3 $${\rm The \:selectivity \:of \:each \:component \:(wt\%) \:is \:calculated \:by \:taking \:the \:yield \:(wt \%) \:divided \:by \:the \:total \:conversion,\: then \:multiplying \:by \:100 }$$

## Result and Discussion

To establish a comprehensive correlation between structural properties and catalytic performance, we systematically characterized the synthesized materials. First, we examined the surface and textural properties using N₂ adsorption–desorption measurements. Next, we conducted XRD analysis to confirm phase purity, and employed TEM imaging to assess particle morphology and Pt dispersion.

### Surface characteristics

**Fig. 2:**
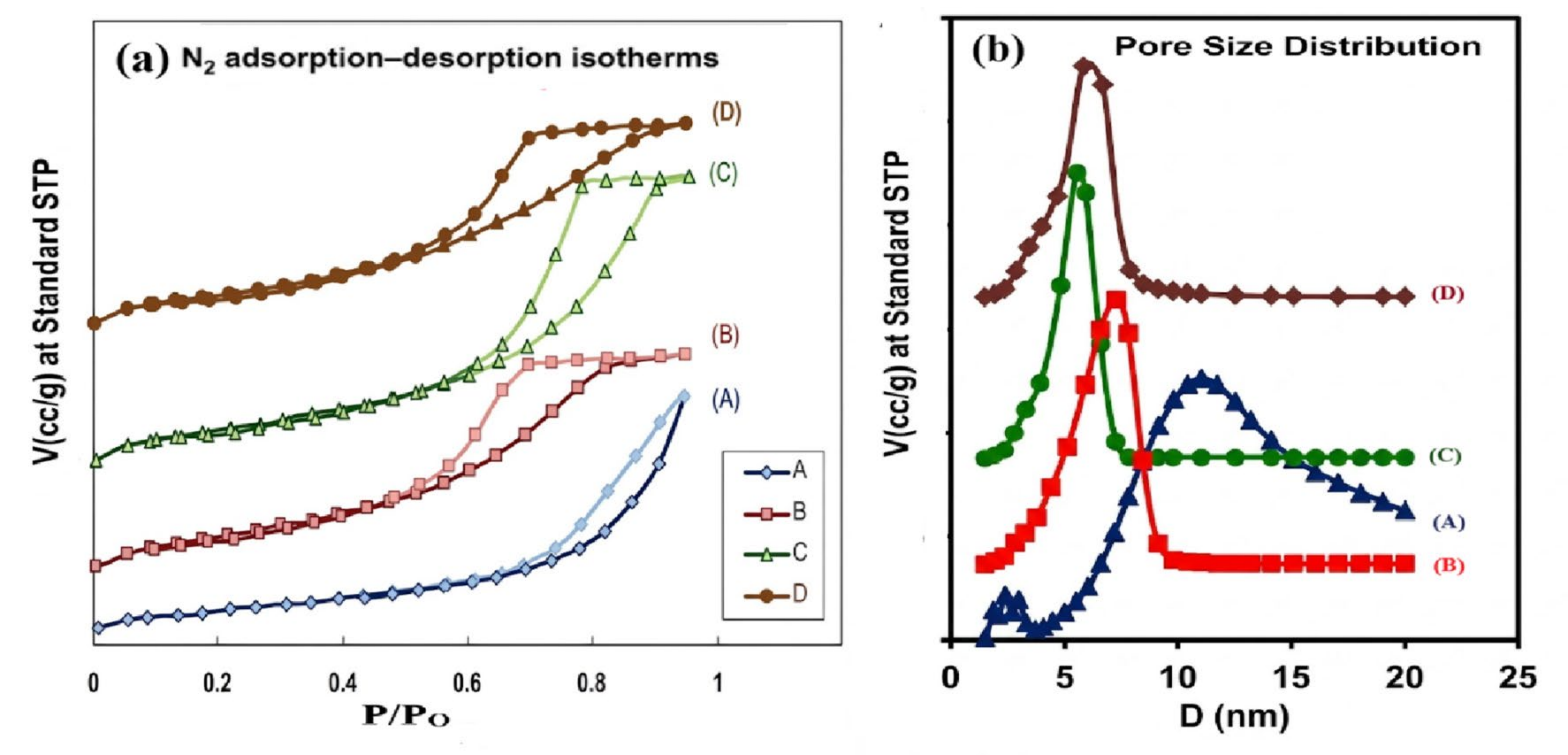
N₂ adsorption-desorption isotherms (**a**) and pore size distribution curves (**b**) for the alumina supports: A (A), AC_1_ (B), AC_2.5_ (C), and AP (D).

The surface characteristics of the synthesized alumina supports were assessed through N_2_ adsorption–desorption isotherms and pore size distribution (PSD) analysis, as illustrated in Fig. 2**(a**,** b).** The corresponding textural parameters are summarized in Table 2. The parent alumina sample (A), Fig. 2**(a)** shows a pseudo-Type II isotherm with an H3 hysteresis loop, indicating the presence of mainly macroporous structures formed by aggregated of plate-like particles that create slit-shaped pores^87,88^.

The PSD curve of parent nano alumina (A) Fig. 2**(b)** showed mixed pore systems centered at 2.29, 2.79, 3.5 and 10.90 nm. The main hydraulic diameter shows a broad pore size distribution centered at 10.90 nm, accompanied by a significant fraction of mesoporous. The polymodal pore size distribution (PSD) curve shown in Fig. 2**(b)** indicates the presence of a mixture of micro or narrower mesoporous and wider mesoporous systems. According to the calculated surface parameters illustrated in Table 2, the synthesized nano alumina (A) exhibited a specific surface area of 140 m^2^g^−1^, total pore volume of 0.43 cm^3^g^−1^ and average pore radius of 10.9 nm. The N₂ adsorption–desorption isotherms obtained from the modified alumina nanopowders, synthesized using surfactant or triblock polymer templates (denoted as AC_1_, AC_2.5_, and AP), display very similar profiles. All isotherms are classified as type IV according to IUPAC standards, which is typical of mesoporous materials. The observed H₂-type hysteresis loop suggests the presence of ink-bottle-shaped pores with narrow necks and wider openings. This specific pore geometry often results in pore-blocking phenomena, where the evaporation of the adsorbed liquid is controlled by the diameter of the pore neck.

The AC_1_ sample showed hysteresis closure at P/P_o_ lattice parameter = 0.5–0.8 while AC_2.5_, by increasing the weight of CTAB surfactant from 1 g to 2.5 g showed the hysteresis closure at higher relative pressure, viz., 0.6–0.9. In contrast, the AP sample displays a hysteresis loop within the range of 0.5 to 0.99 P/Po. It has been suggested that the lower closure point is largely unaffected by the porous characteristics of the materials. Instead, the lower limit of the hysteresis loop is determined by the tensile strength of the capillary condensed liquid nitrogen. It depends also on the geometry of the pores and the temperature. The PSD curves for the modified alumina samples, AC_1_, AC_2.5_ and AP Fig. 2**(b)** showed unique, more controlled and organized pore system and the mean hydraulic diameter was centered at 7.6, 5.8, and 6.6 nm, respectively. The AC_2.5_ sample displayed narrower pore size distribution than that of AC_1_ due to increasing the amount of CTAB, i.e., the increase of CTAB seemed to lead to more regulation of pores with better distribution profile. The pore size diameter for AC_1_ sample is clearly detected in PSD curve Fig. 2**(b)**, centred at 7.6 nm which shifted to the lower mesopore diameter value at 5.8 nm for AC_2.5_ sample.

**Table 2:** Surface parameters and average crystallite size calculated from XRD for A, AC_1_, AC_2.5_ and AP supports.

Support	S _BET_ (m^2^ g^− 1^)	V_*P*_ (cm^3^ g^− 1^)	D_*p*_ (nm)	ao (nm)^a^	Crystallite size(nm)		
					**D**_**XRD**_^**b**^	**D**_**BET**_^**c**^	**D**_**TEM**_^**d**^
**A**	140	0.43	10.9	0.119	8.3	10.8	0.8–1.2
**AC**_**1**_	243	0.52	6.5	0.118	11.6	6.2	1.8–3.5
**AC**_**2.5**_	285	0.63	7.7	0.118	12.1	5.3	1.5–3.5
**AP**	228	0.39	5.7	0.118	10.3	6.6	1.5–5.2

**a**: is the unit cell parameter of alumina lattice calculated by equation.

**a**= d440 [j^2^+ k^2^+ l2]^1/2^ **(4)**.

**b**: crystallite size calculated by scherrer equation.

**c**: crystallite size calculated by BET equation.

Table 2 summarizes the surface parameters of the synthesized alumina supports with various modifiers. The specific surface areas (S_BET_) of A, AC_1_, AC_2.5_ and AP samples were 140, 243, 285, and 228 m^2^g^−1^, respectively. The total pore volume were 0.43, 0.52, 0.63, and 0.39 cm^3^g^−1^, respectively. The specific surface area and total volume of the neat alumina support increase with the addition of CTAB surfactant molecules. In contrast, the specific surface area and total pore volume of the AP support prepared with pluronic (P123) are lower than those of the CTAB-prepared support. The N₂ adsorption–desorption isotherms for all samples display type IV behavior as classified by IUPAC, which confirms their mesoporous characteristics. The observed hysteresis loop is linked to capillary condensation within the mesopores and indicates variations in pore structure and connectivity among the samples. The particle size (D_BET_) was estimated from the BET surface area using the formula D_BET_ = 6/(ρ_SBET_), where ρ is the theoretical density of the powder (3.97 g/cm³ for Al_2_O_3_).assuming spherical geometry and uniform surface accessibility. This calculated D_BET_ represents an equivalent particle size related to the external surface area of particles or agglomerates, rather than the actual crystallite size.

It’s important to understand that DBET is linked to the external surface area of particles or agglomerates and does not directly correspond to the crystallite size determined by X-ray diffraction (XRD). In contrast, the XRD crystallite size reflects the size of coherent diffraction domains, which are individual crystalline regions. Consequently, the differences between XRD crystallite size and BET-derived particle size stem from their distinct measurement principles: XRD measures the size of coherent crystalline domains, while BET-derived values are affected by particle aggregation and surface accessibility, excluding contributions from closed or inaccessible pores. Therefore, both parameters should be seen as complementary rather than directly comparable.

**Fig. 3:**
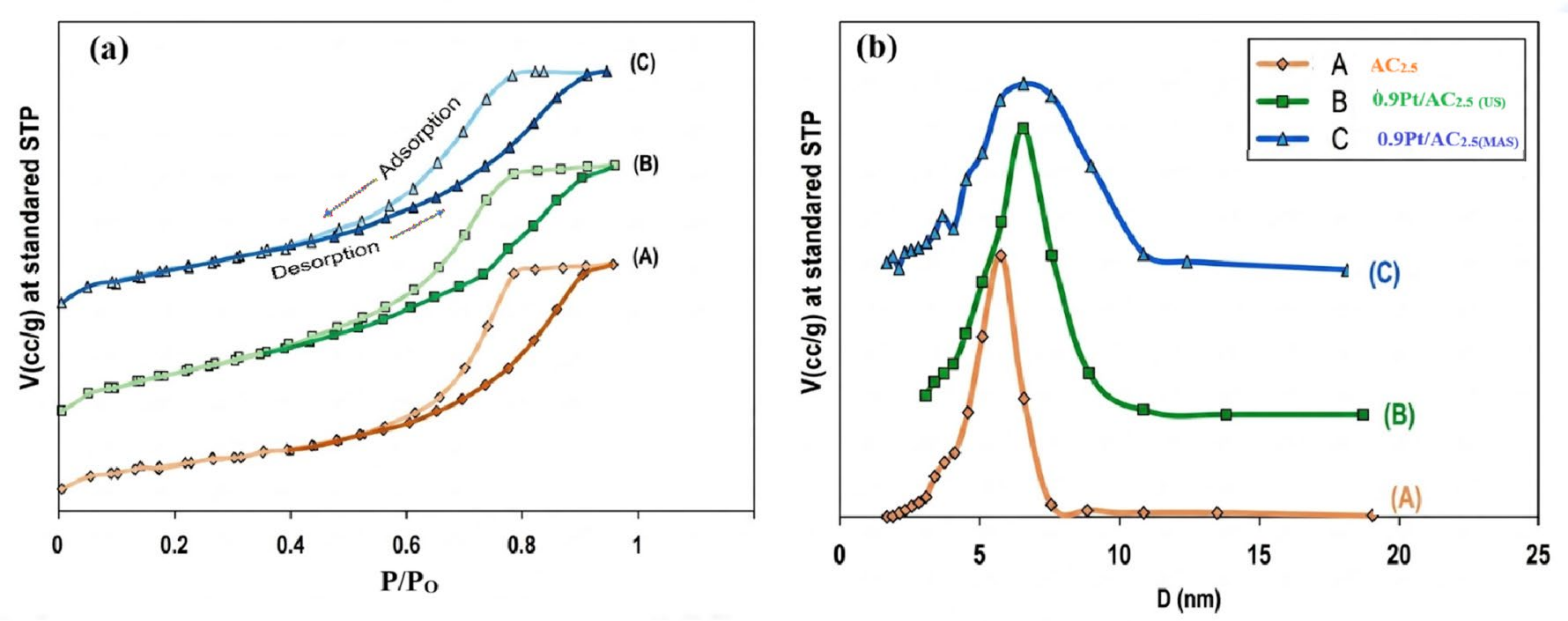
N₂ adsorption-desorption isotherms (**a**) and pore size distribution curves (**b**) for AC_2.5_ nanosupport (A), 0.9 wt% Pt/AC_2.5 (US)_ (B), and 0.9 wt% Pt/AC_2.5 (MAS)_ (C).

Additionally, 0.9 wt% Pt loading was supported on the surface of alumina (AC_2.5_) prepared with CTAB as the surfactant. The reduction step involved either ultrasonic (US) or microwave assisted solution radiation methods (MAS) showed in Fig. 3**(a**,** b)**. The choice of this AC_2.5_ support was acknowledged to the high surface area, high thermal stability, narrower pore size distribution as well as particle size distribution. Therefore, the various physico-chemical characteristics of these two samples will be investigated. It is clear in **Figsure 3(a)** that, the obtained adsorption isotherms for the supported 0.9 wt% Pt/AC_2.5_ reduced by ultrasonic and microwave methods, are very close to that of the modified alumina support (AC_2.5_) probably due to the nanosize of supported metal. All samples fall under type IV according to IUPAC classification, displaying an H2 hysteresis loop in the P/Po range of 0.6–0.9, which is characteristic of mesoporous materials. These materials feature ink-bottle type pores, characterized by narrower openings leading to broader inner sections. The surface parameters obtained from the adsorption isotherms are summarized in Table 3.

**Table 3:** Surface parameters of neat support Al_2_O_3_ and optimum of Pt-AC_2.5_ nanocatalysts by US and MAS method.

Catalyst	S _BET_ (m^2^ g^− 1^)	V_*P*_ (cm^3^ g^− 1^)	*r*_*p*_ pore radius (nm)
**Alumina (AC**_**2.5**_ **)**	285	0.63	7.7
**0.9 wt% Pt/AC**_**2.5(US)**_	364	0.61	6.7
**0.9 wt% Pt/AC**_**2.5(MAS)**_	344	0.58	6.8

The support of Pt nanoparticles, achieved through ultrasonic (US) and microwave-assisted (MAS) reduction methods, leads to an increase in the surface area of the AC_2.5_ support, accompanied by a decrease in pore dimensions (VP and rp). This increase in surface area is particularly significant for the supported Pt nanocatalyst (0.9 wt% Pt/AC_2.5_), especially when the ultrasonic reduction method is applied, as shown in Fig. 3**(b)**. For the 0.9 wt% Pt/AC_2.5_ nanocatalyst reduced using the ultrasonic method, a wider pore size distribution curve is observed compared to that of the pure AC_2.5_ support, with a noticeable shift in the hydraulic radius from approximately 6 nm to 7 nm. Here, rₚ denotes the pore radius, while the pore size distribution (PSD) curves in Fig. 3 are presented in terms of pore diameter. It is important to note that the observed changes in surface area and textural properties may not be solely due to Pt incorporation. The MAS and US treatments can also affect the pore structure and surface characteristics of the support, even without Pt. Consequently, the improvement in surface properties may stem from the combined effects of the preparation method and metal incorporation. For the same supported Pt sample reduced by microwave method, more widening in pore size distribution is occurred with almost the same most probable hydraulic radius (~ 7 nm) together with a small fraction of somewhat narrower pores of ~ 3 nm. This may be related to the transfer of a fraction of Pt nanoparticles from inner to outer surface. The higher surface area and enhanced pore structure are anticipated to improve reactant diffusion and accessibility to active sites, thereby positively impacting catalytic performance.

### XRD analysis

A transparent gel-like precursor containing aluminum cations was created by mixing ammonium bicarbonate and aluminum nitrate solutions in deionized water at a pH of approximately 7.5–8.5. The mixture was continuously stirred and maintained at 70 °C. The primary reactions involved in this preparation can be summarized as follows:

4 $${\rm NH_{4}HCO_{3} + H_{2}O \rightarrow\: NH4OH + H_{2}O + CO_{2}}$$

5 $${\rm Al(NO_{3})_{3} + 3NH_{4}OH \rightarrow\: Al(OH)_{3} \downarrow\: + 3NH_{4}NO_{3}}$$

7 $${\rm Al(OH)_{3} \rightarrow \:AlOOH \downarrow\: + H_{2}O}$$

8 $${\rm 2AlOOH \rightarrow\: Al2O3 + H_{2}O}$$

The hydrolysis of ammonium bicarbonate in an aqueous medium generates OH⁻ ions through reaction (5). The precipitate formed in reaction (6) was aged at approximately 70 °C to enhance gel homogenization via a slow ripening process. This aging step is essential for converting amorphous Al(OH)₃ into crystalline boehmite (AlOOH), as shown in reaction (7). The resulting precipitate was washed multiple times with deionized water, followed by ethanol, to remove residual ionic impurities such as sodium. Gamma-alumina (γ-Al₂O₃) was obtained by calcining the dried boehmite at 550 °C in air, according to reaction (8)^89^.

**Fig. 4:**
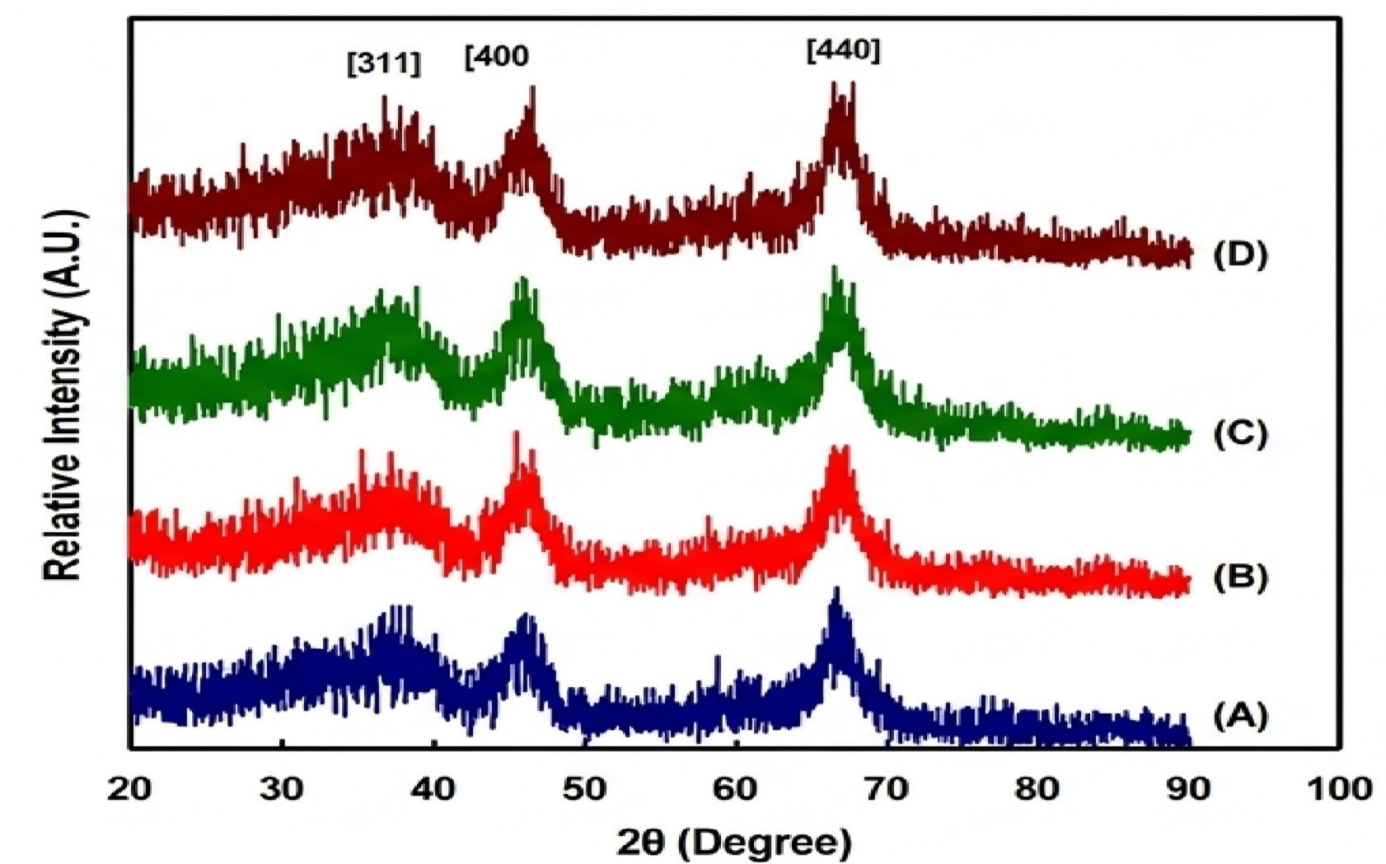
XRD of alumina supports: parent alumina support A (A), AC_1_ (B), AC_2.5_ (C) and AP (D).

Figure 4 (A–D) displays the XRD patterns of the as-dried boehmite samples that were calcined at 550 °C. The XRD diffractograms for samples A, AC1, AC_2.5_, and AP confirm the formation of γ-Al₂O₃ with a cubic spinel lattice (space group *Fd3m*) (JCPDS No. 29–0063). Characteristic diffraction peaks appear at 2θ = 37.5^°^ (311), 45.7^°^ (400), and 66.7^°^ (440), indicating a defect cubic spinel structure with vacancies at Al^3^⁺ positions. Each unit cell contains 32 oxygen atoms and 64/3 Al^3^⁺ ions, maintaining stoichiometric balance. Aluminum cations occupy both octahedral and tetrahedral sites, though their relative occupancy is still under discussion. The XRD patterns indicate that the synthesized alumina supports are highly crystalline and exhibit nanoscale features, evidenced by the broadening of diffraction peaks due to the presence of small crystallites. The average crystallite size was estimated using the Scherrer equation.

9 $$D= K\lambda/ \upbeta \:cos\: \theta$$

where K is the shape factor (~ 0.9), λ is the X-ray wavelength, β is the full width at half maximum (FWHM) of the diffraction peak, and θ is the Bragg angle.

10 $${\rm n \lambda =2d \:sin\:\theta}$$

The interplanar spacing (d) was calculated using Bragg’s law. Due to the complex defective spinel structure of γ-Al₂O₃, the lattice parameters were estimated using standard crystallographic approximations.The calculated crystallite sizes for samples A, AC_1_, AC_2.5_, and AP are summarized in Table 2. Thus, by precisely controlling synthesis parameters such as temperature, pH, and aging conditions, nano-sized γ-Al₂O₃ can be effectively produced. It is shown that the A sample having the smallest crystallites size (8.3 nm) among all synthesized nano-alumina under this investigation. The unit cell lattice parameters were calculated to be 0.1192, 0.1185, 0.1186, and 0.1187 nm for samples A, AC_1_, AC_2.5_, and AP, respectively, indicating that the modification did not significantly alter the defective cubic structure of γ-Al₂O₃^22,90^.

**Fig. 5:**
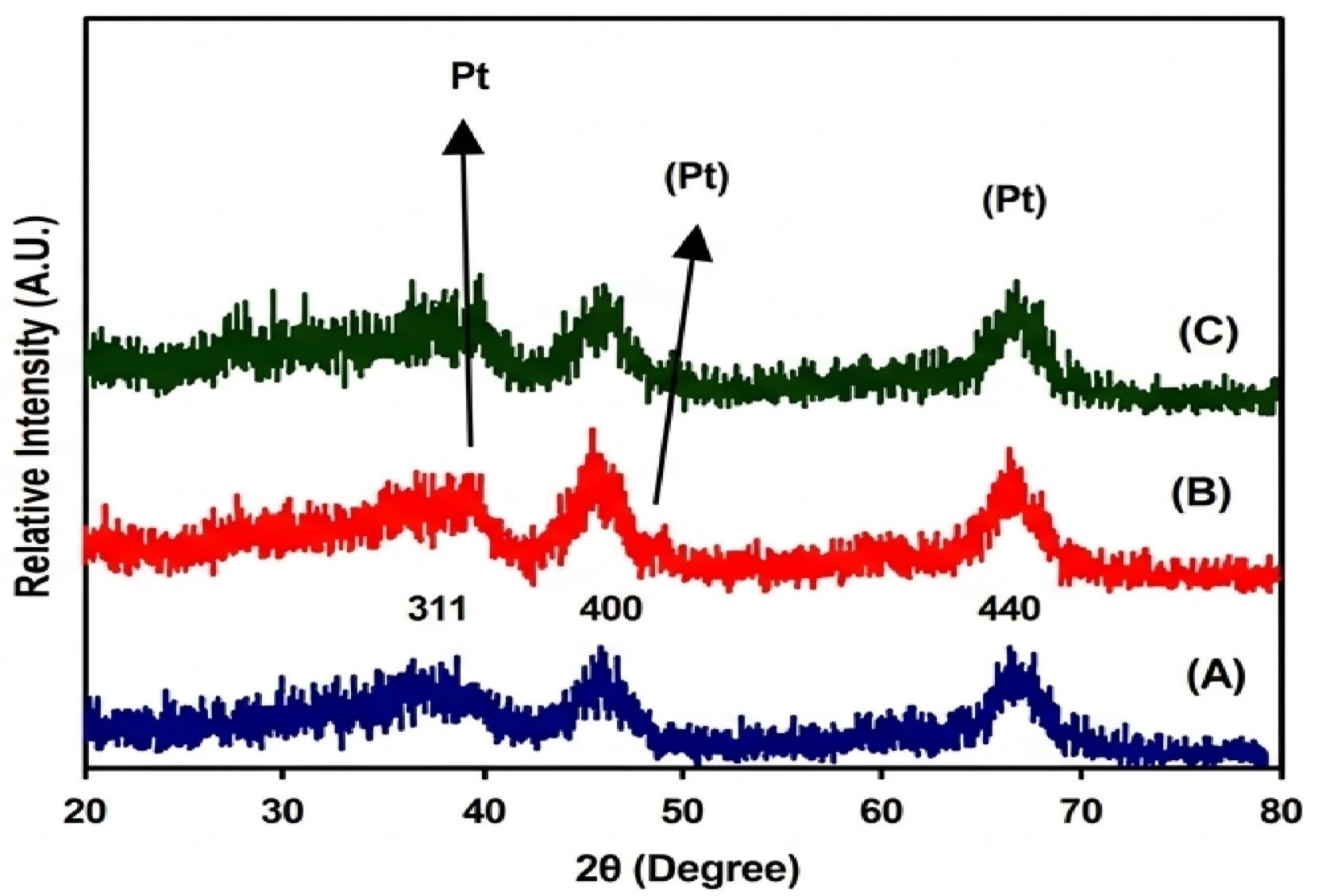
XRD of AC_2.5_ (A), 0.9 wt% Pt/AC_2.5_ (_US_), (B) and 0.9 wt% Pt/AC_2.5_ (_MAS_) (C).

Figure 5 (A-C) presents the XRD patterns of in situ reduced Pt-based γ-Al_2_O_3_ (AC_2.5_) nanocatalysts. The XRD pattern for the AC_2.5_ support indicates the presence of the γ-Al_2_O_3_ phase, which exhibits a cubic spinel lattice (space group Fd3m), as referenced in (JCPDS No. 29–0063) as evidenced from the intense three lines at 2 θ of 37.5 (311), 45.7 (400) and 66.7 (440). The characteristic features of AC_2.5_ nano-support are maintained in the synthesized 0.9 wt% Pt/AC_2.5_ nanocatalyst reduced by US and MAS methods. For supported Pt catalyst sample, new diffraction lines at small diffraction lines were detected at 39.8^◦^ (1 1 1), 46.5^◦^ (2 0 0) and 67.8^◦^ (2 2 0), attributed to a Pt metallic phase according to (JCPDS No. 01–1190). The absence of sharp Pt peaks is due to the low metal loading (0.9 wt%) and the high dispersion of Pt nanoparticles, which fall below the detection limit of XRD. This further confirms the effectiveness of the MAS and US reduction methods.

### Thermal analysis

**Fig. 6:**
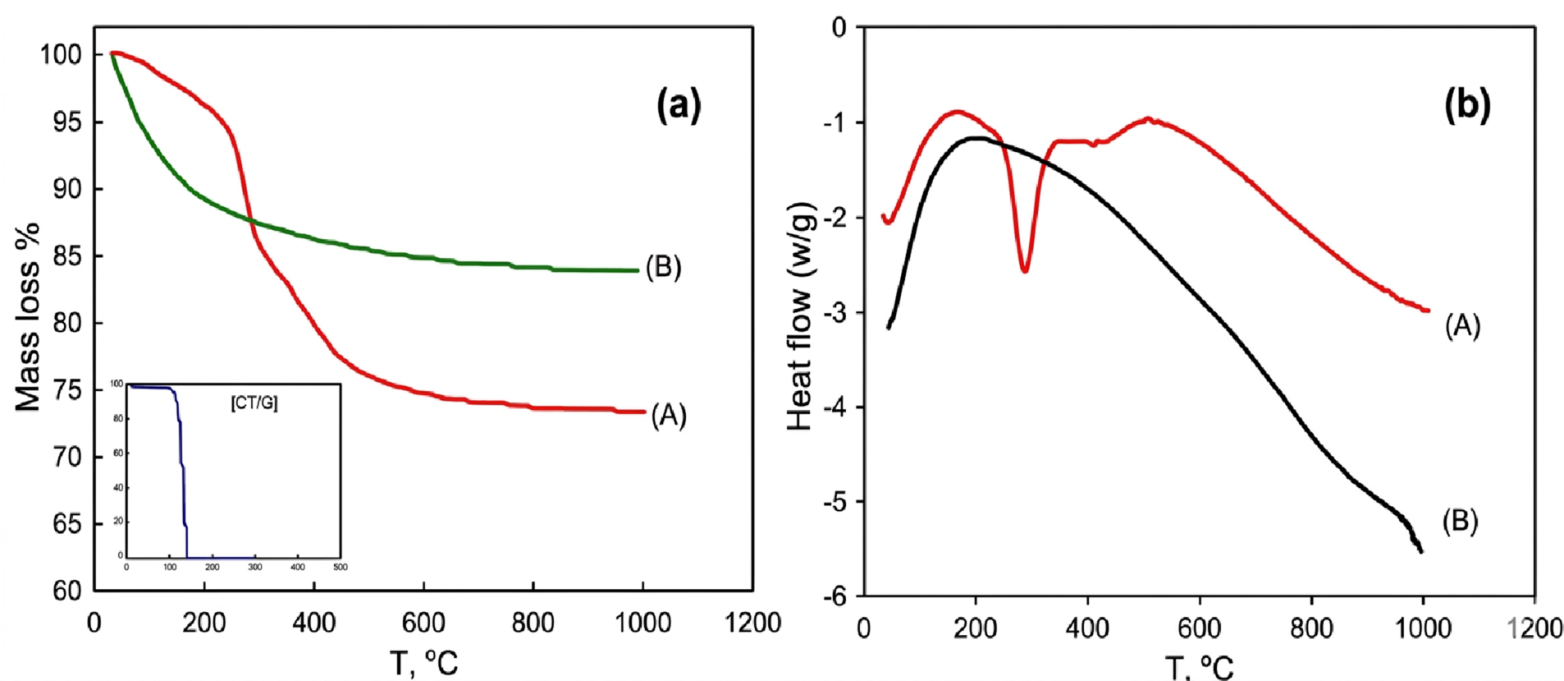
TGA (a) and DSC (b) of the alumina precursor prior to calcination (A) and AC_2.5_ (B).

The TGA curve of alumina precursor before calcination Fig. 6**(a)** shows two major mass loss, viz., in the temperature ranges: 90 ~ 300^°^ C (14.2%) and ˃300^°^ C ~ 550^°^ C (~ 11.0%). At temperature up to 70 °C, the obtained precipitate is most probably bayerite (reaction 2). The initial region of mass loss due to endothermic processes at (90–300 °C), represents the removal of solvent molecules, involving primarily water, ethanol, and acetone at 90–100 °C together with the start of decomposition of CTAB (associated with DSC peak at 106^°^ C). The transformation of bayerite (Al(OH)_3_) to boehmite (AlOOH) (reaction 3) seems to take place up to 300^°^ C, with complete decomposition of CTAB molecules. This can be associated with the marked endothermic peak at ~ 300^°^ C refer that also associated to the second DSC peak of CTAB at 280^°^ C. In the second endothermic mass loss region (˃300 ~ 550^°^C), the boehmite evidently transforms into γ- Al_2_O_3_ according to (reaction 4), as associated with the relatively weak endothermic peak at 400^°^ C related to evolution of H_2_O molecules. On the other hand, the TGA curve of alumina (AC_2.5_) Fig. 6**(a)**, its mass loss appears to occur below 100^°^ C that can be attributed to some volatilization of water. This can be confirmed by some broad DSC endothermic peak in this region Fig. 6**(b)**. These findings in general reflect the relatively high thermal stability of the synthesized alumina nanosupport (AC_2.5_). A quantitative summary of the mass loss over the different temperature ranges for the studied samples is presented in Table 4.

**Table 4:** Mass loss from thermal analysis of various alumina supports.

Temp. rang	A	AC_1_	AC_2.5_	AP
**≤ 200**	10.63	10.63	11.36	10.1
**200–400**	3.04	3	2.99	2.75
**400–600**	1.44	1.49	1.4	1.37

**Fig. 7:**
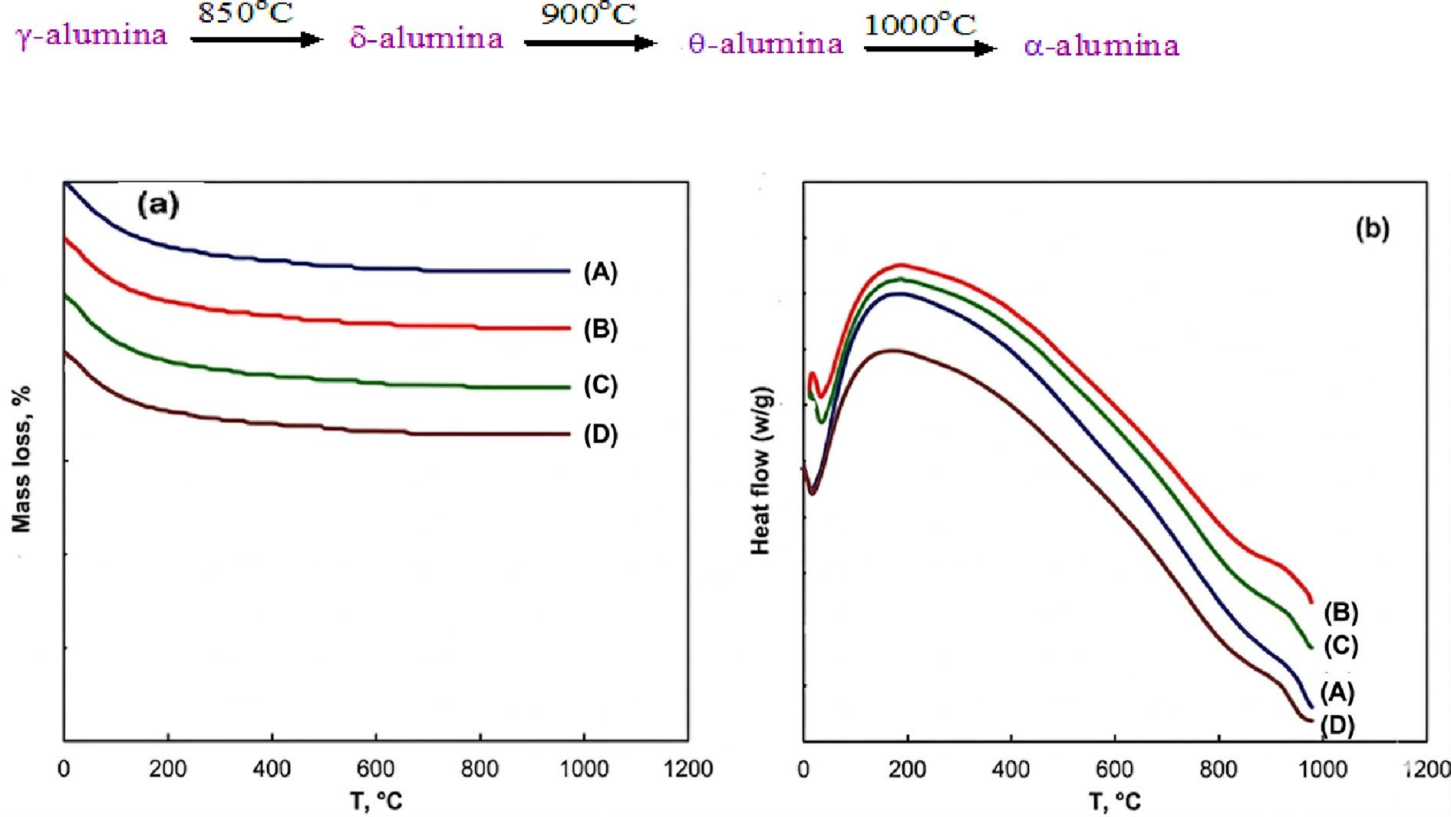
TGA (a) and DSC (b) of alumina support: parent alumina support A (A), AC_1_ (B), AC_2.5_ (C) and AP (D).

Figure 7(a, b) showed that TGA and DSC analyses of all synthesized alumina nanocatalysts under this investigation (A, AC_1_, AC_2.5_ and AP). Both TGA and DSC of A, AC_1_, AC_2.5_ and AP samples showed the same thermal trend and demonstrated excellent thermal stability up to 1000 °C. The endothermic peak observed at temperatures below 100 °C indicates the removal of physically adsorbed water from the catalyst’s surface in all cases (Fig. 7a). Table 4 revealed The TGA of all nano- alumina supports prepared by sol gel method. One can be recalled that there is no sign of more mass loss in TGA curves, which is most likely related to successive and complete decomposition of precursor. The DSC curve (Fig. 7b) also displays a broad exothermic peak above 900 °C, which is associated with the dehydroxylation of residual hydroxyls in the alumina lattice and the transformation of γ-alumina to the θ-alumina phase of the support. This behavior aligns with the reported phase transformation of aluminum hydroxide precursors into γ-Al_2_O_3_, as described in the literature^91,92^.

**Table 5:** Mass loss (%) from thermal analysis of AC_2.5_ nanosupport and 0.9 wt% Pt/AC_2.5_ reduced by ultrasound and microwave-assisted synthesis methods.

Temperature range (°C)	AC_2.5_	0.9 wt% Pt/AC_2.5 (US)_	0.9 wt% Pt/AC_2.5 (MAS)_
≤ 200	10.36	10.52	22.44
200–400	2.98	3.74	3.90

**Fig. 8:**
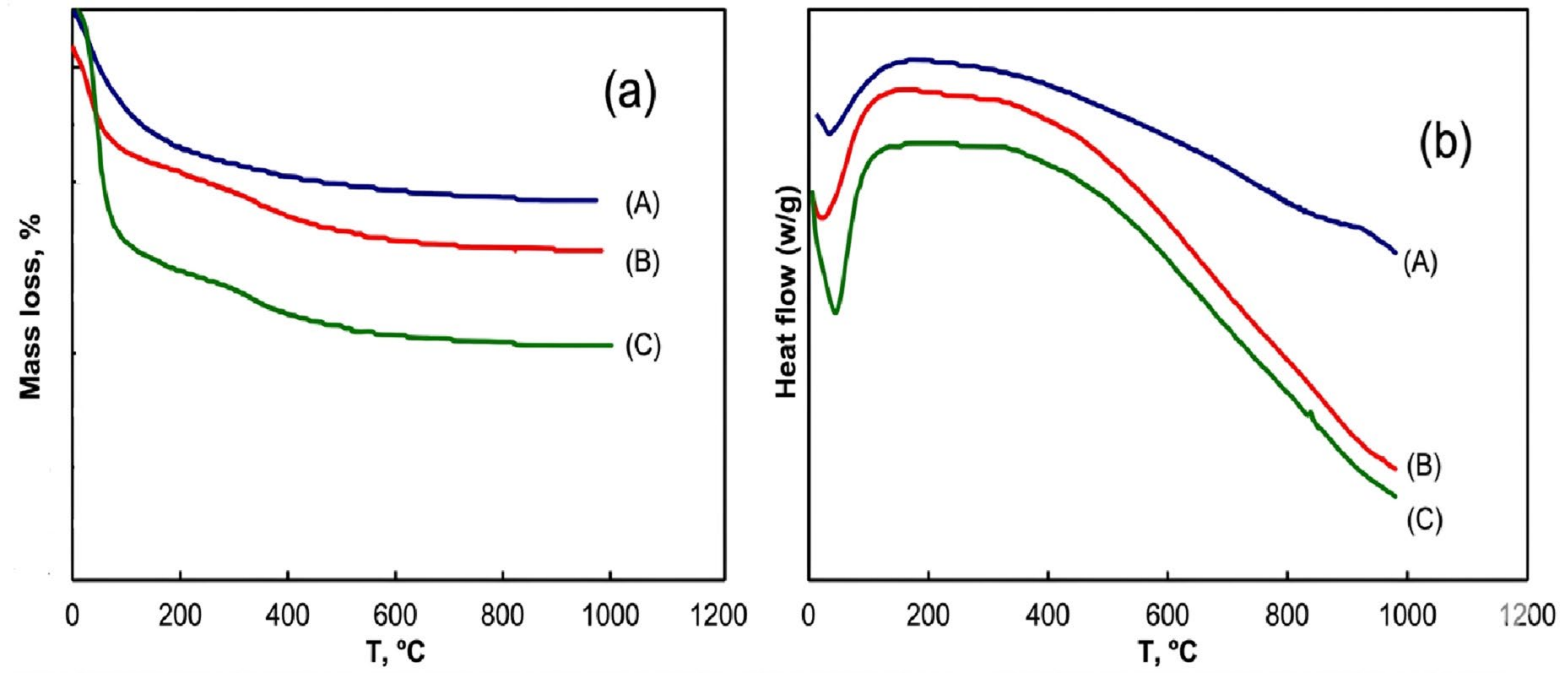
TGA and DSC of (A) AC_2.5_, (B) 0.9 wt% Pt/AC_2.5_ (_US_) and (C) 0.9 wt% Pt/AC_2.5_ (_MAS_).

The Differential Scanning Calorimetry (DSC) and Thermogravimetric (TG) profiles were obtained from 25^°^C to 1000^°^C under a nitrogen atmosphere for the support (AC_2.5_) and the selected nanocatalysts, 0.9 wt% Pt/AC_2.5_ reduced by US and MAS methods are represented in Fig. 8**(a**,** b)**. **Table 5** summarizes the quantitative mass loss values for AC_2.5_ nanosupport and Pt/AC_2.5_ nanocatalysts, which were reduced using US and MAS methods.

The major mass loss is occurred below 200^°^ C, accompanied with endothermic peaks centered below ~ 100^°^ C related to evaporation of the physically adsorbed water. Another mass loss step is clearly observed within the range 200–400^°^C for 0.9 wt% Pt/AC_2.5_ (_US_) and 0.9 wt% Pt/AC_2.5_ (_MAS_) nanocatalyst samples reducted by MAS or US methods Fig. 8 being related probably to the removal of residual water of hydration and progressive decomposition of precursor and other remnants. This seems not accompanied with heat changes i.e. without endothermic peaks. The results generally reflect the relatively high thermal stability of both Pt/AC_2.5_ nanocatalysts reduced by ultrasonic and microwave methods up to 1000^°^ C.

### TEM micrographs

**Fig. 9:**
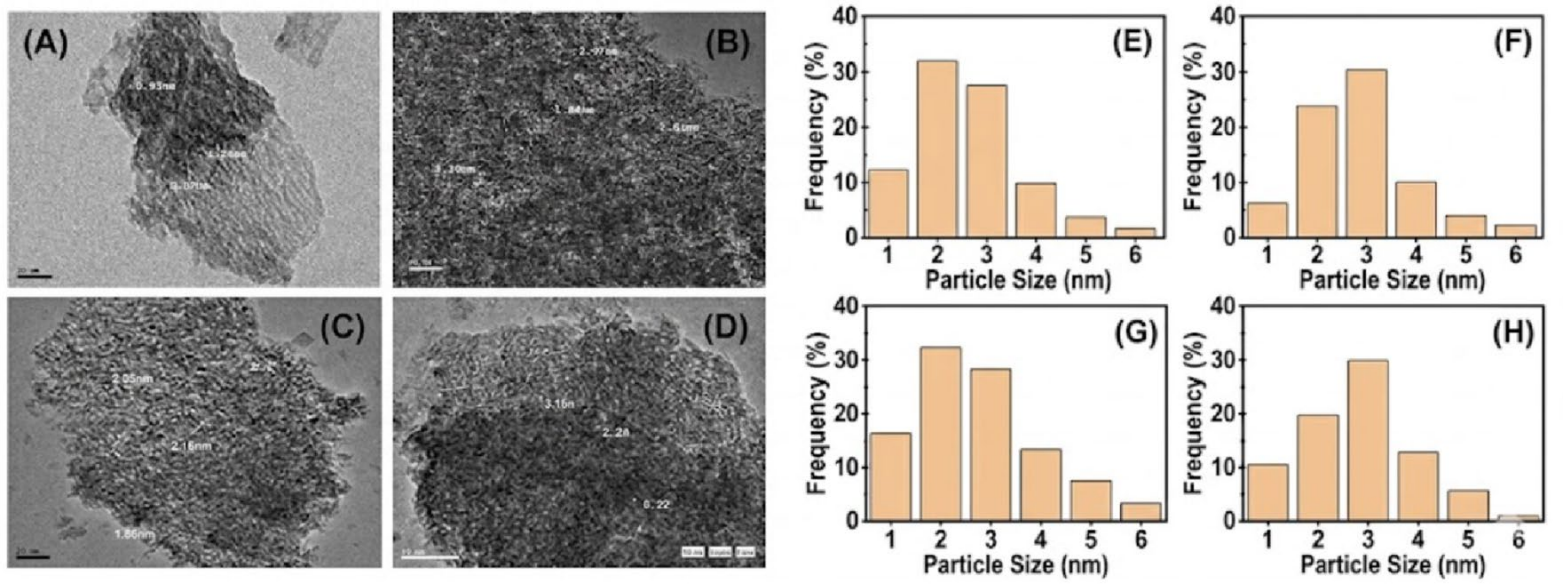
TEM micrographs and particle size distribution histograms of γ-Al₂O₃ support samples (A, H), AC_1_ (B, F), AC_2.5_ (C, G) and AP (D, H).

A particle size distribution histogram was created by measuring at least 50 nanoparticles from the TEM images, allowing for a more accurate evaluation of the particle size distribution. Figures 9**(A-D)** display TEM micrographs of various γ-Al_2_O_3_ nanopowders, which were produced by calcining as-dried boehmite precursors with different modifications. The micrographs of all alumina samples reveal fine spherical particles. To provide a more quantitative assessment, particle size distribution histograms were constructed for each sample, as shown in Fig. 9**(E-H).** The histograms indicate that the average size of the nanoparticles.The statistical analysis indicates that the particle size of the γ-Al₂O₃ support ranges from 0.87 to 5.5 nm, confirming that the majority of the support particles are within this nanometric range.

**Fig. 10:**
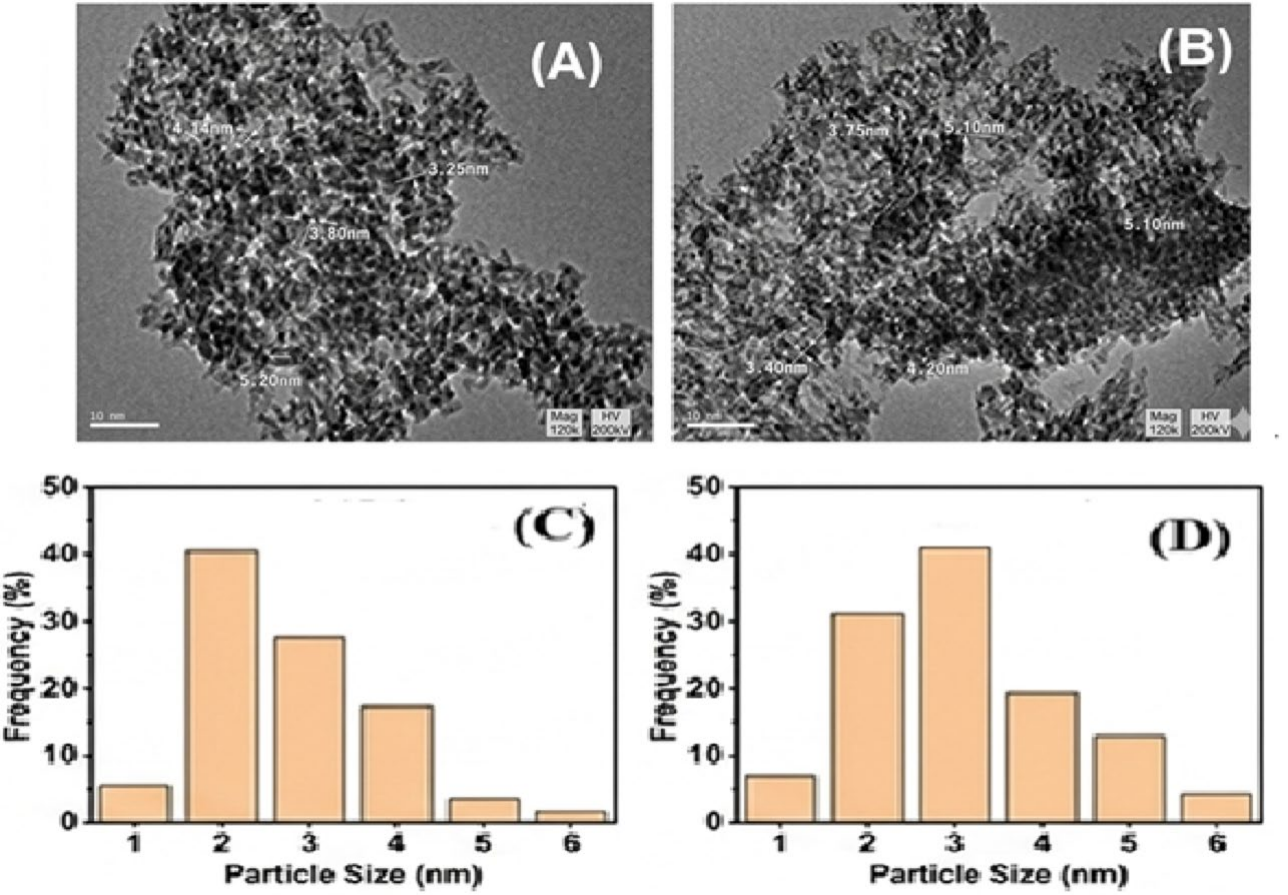
TEM micrographs and particle size distribution histograms of 0.9 wt% Pt/AC_2.5_ catalysts prepared by US (A, C) and MAS (B, D) methods.

The TEM micrographs of the AC_2.5_ support and 0.9 wt% Pt/AC_2.5_ nanocatalysts reduced using ultrasonic (US) and microwave (MAS) techniques are illustrated in Fig. 10. In accordance with the reviewer’s recommendations, high-resolution images and corresponding size distribution histograms are provided to verify the catalyst morphology. The TEM micrographs of both supported Pt/AC_2.5_ catalysts (images A and B) show homogeneous and well-distributed Pt nanoparticles. The histograms in Fig. 10**(C-D)** indicate that the average size of the Pt nanoparticles ranges from 3 to 5 nm for the 0.9 wt% Pt/AC_2.5 (US)_ and from 5 to 6 nm for 0.9 wt% Pt/AC_2.5 (MAS)_ nanocatalysts. These observations and the quantitative data derived from the histograms confirm the effectiveness of both ultrasonic and microwave irradiation as reduction methods for producing stable, dispersed platinum nanoparticles with average sizes not exceeding 6 nm.

The smaller size range of 0.87–5.5 nm corresponds to the alumina support particles, while the larger size range of 3–6 nm is attributed to the supported Pt nanoparticles.

Although the Pt nanoparticles in the US catalyst are slightly smaller (3–5 nm) than those in the MAS catalyst (5–6 nm), the MAS catalyst demonstrates better catalytic performance. This improvement is due to stronger metal–support interactions and more efficient use of active sites, rather than particle size alone.

### Analysis of H_2_-Chemisorption

To quantitatively evaluate the active metallic phase and the degree of reduction, H_2_-chemisorption measurements were performed at ambient temperature. The dispersion parameters obtained for the 0.9 wt% Pt/AC_2.5_ catalysts prepared by various methods are summarized in Table 6.

**Table 6:** H₂ chemisorption results of 0.9 wt% Pt/AC_2.5_ catalysts, including metal dispersion, average Pt crystallite size, and metal surface area.

Catalyst	Metal Dispersion (D)	Avg. Pt Crystallite Size (nm)	Surface Area (m^2^/g metal​)
**0.9 wt% Pt/AC**_**2.5 (US)**_	0.405	0.705	399.72
**0.9 wt% Pt/AC**_**2.5 (MAS)**_	0.340	0.826	338.51

The results demonstrate that both preparation methods produced catalysts with high metal dispersion. The 0.9 wt% Pt/AC_2.5 (US)_ catalyst displayed the highest dispersion (D = 0.405) and a smaller average Pt crystallite size of 0.705 nm. This contributed to a greater metallic surface area of 399.72 m²/g metal and an increased number of accessible active sites. In comparison, the 0.9 wt% Pt/AC_2.5(MAS)_ catalyst exhibited slightly lower dispersion (0.340) and a larger crystallite size of 0.826 nm. It is important to note that the Pt crystallite sizes obtained from H₂ chemisorption are smaller than those observed via TEM, which range from 3 to 6 nm. This discrepancy arises from the different measurement principles: H₂ chemisorption estimates particle size based on accessible surface metal atoms and dispersion, while TEM provides direct visualization of the physical size of particles, including larger ones. Consequently, the results from these two techniques are not directly comparable. This results confirm the effective reduction of Pt species to their metallic state and elucidate the observed catalytic performance.

### Catalytic activity of selected catalysts

Before discussing catalytic performance, it is important to clarify that γ-Al₂O₃ primarily serves as a support material and exhibits very limited intrinsic catalytic activity under the reaction conditions studied. In this investigation, the bare support exhibited very low conversion in the n-hexane reaction, with no detectable formation of aromatic products. Consequently, the observed catalytic performance is mainly due to the presence of Pt active sites. Moreover, the alumina support significantly enhances Pt dispersion and provides surface acidity, which together facilitate bifunctional dehydrogenation and cracking pathways^80^. These findings underscore the importance of the synergistic interaction between the metal and support in both MAS and US catalysts. Additionally, the 0.9 wt% Pt/AC_2.5 (MAS)_ catalyst demonstrated stable catalytic performance without significant deactivation, attributed to the preservation of its mesoporous structure and the strong metal–support interaction, as confirmed by TEM analysis. Although the catalysts were subjected to MAS and US treatments, along with subsequent washing steps, a slight possibility of Pt loss cannot be entirely ruled out. However, the consistent catalytic performance and similar trends observed across the samples, along with the lack of significant variations in activity, indicate that any potential changes in Pt loading are negligible. This conclusion is further reinforced by quantitative H_2−_ Chemisorption results and TEM analysis, which demonstrate high Pt dispersion and strong metal–support interactions. Therefore, any minor Pt loss, if it occurs, does not significantly affect the overall catalytic behavior or the comparative evaluation of the catalysts.

#### Cyclohexane conversion

**Fig. 11:**
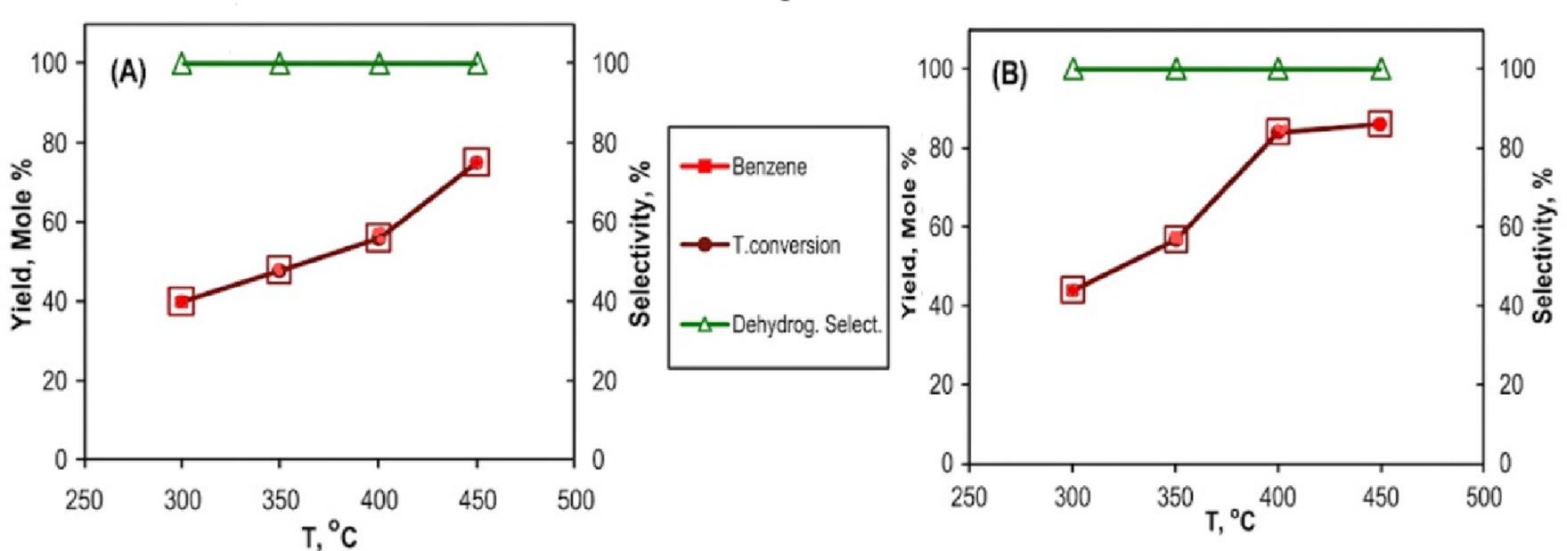
Catalytic conversion of cyclohexane was performed using 0.9 wt% Pt/AC_2.5_ nanocatalysts prepared through two different methods: 0.9 wt% Pt/AC_2.5 (US)_ (A) and 0.9 wt% Pt/AC_2.5 (MAS_) (B).

The catalytic conversion of cyclohexane using the 0.9 wt% Pt/AC_2.5_ catalyst samples, reduced by the US and MAS methods, is illustrated in Fig. 11; Table 7. The results indicate that total conversion increases with rising reaction temperatures, ranging from 250 to 450^°^C. Additionally, the yield of benzene rises with temperature, reaching 75% at 450^°^C for the 0.9 wt% Pt/AC_2.5 (US)_ catalyst and 86% at the same temperature for the 0.9 wt% Pt/AC_2.5 (MAS)_ catalyst. The selectivity for benzene was 100% across the entire temperature range for both catalysts, indicating no detectable by-products. The difference observed between selectivity and conversion rates (80–90%) is due to the presence of unreacted cyclohexane, with no detectable by-products under the studied conditions. The result indicated that the 0.9 wt% Pt/AC_2.5 (MAS)_ is the most active for cyclohexane dehydrogenation than 0.9 wt% Pt/AC_2.5_ (_US_). The catalytic results from various reactions demonstrate consistent trends, which support the reliability of the experimental data and confirm the relationship between catalyst structure and performance.

The selected Pt loading (0.9 wt%) was based on previous studies reported in the literature^80^, which demonstrated effective catalytic performance with similar loadings on various supports, including commercial alumina and silica-based systems. In the present study, this loading was applied without performing a systematic optimization of the Pt content. The catalytic performance of the 0.9 wt% Pt/AC_2.5_ catalyst can be attributed to several factors. First, the selected Pt loading contributes to good nanoparticle dispersion, resulting in a greater number of active sites for the reaction. This is further enhanced by the interaction of platinum with defective octahedral sites in the alumina support, which are known for their high activity and ease of reduction. Additionally, the catalyst’s nanostructure is designed to promote strong interactions with surface hydroxyl groups, further stabilizing the platinum and boosting its reactivity and at loading 0.9 wt% Pt/AC_2.5_ achieves the optimal electron density and d-band center position, which enhances C–H bond activation and stabilizes dehydrogenated intermediates without causing over binding. The 0.9 wt% Pt/AC_2.5_ catalyst prepared using the microwave method outperforms the one produced via ultrasonic synthesis. The microwave synthesis process allows some platinum nanoparticles to be positioned outside the pore system, promoting a more uniform dispersion of nanoparticles and a deeper incorporation of platinum into the alumina support, ultimately leading to improved catalytic activity and selectivity in dehydrogenation reactions.

#### n-hexane Conversion

**Fig. 12:**
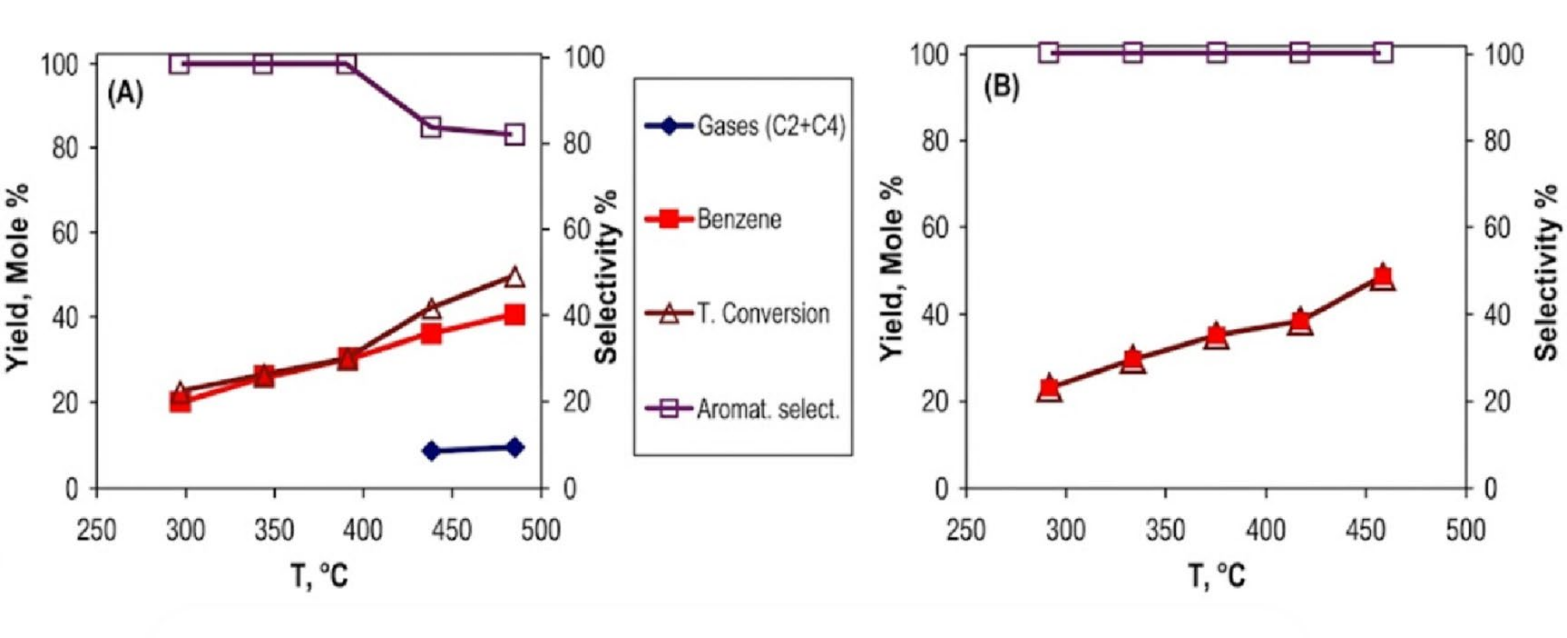
Catalytic conversion of n-hexane was performed using 0.9 wt% Pt/AC_2.5_ nanocatalysts prepared through two different methods: 0.9 wt% Pt/AC_2.5 (US)_ (A) and 0.9 wt% Pt/AC_2.5 (MAS_) (B).

The catalytic conversion of n-hexane was evaluated using 0.9 wt% Pt/AC_2.5_ catalysts prepared by the US and MAS methods over a temperature range of 250–450 °C, as illustrated in Fig. 12. The 0.9 wt% Pt/AC_2.5 (US)_ catalyst achieved a conversion rate of 49% and a benzene yield of 40%, which can be attributed to its higher platinum dispersion and surface area. In contrast, the 0.9 wt% Pt/AC_2.5 (MAS)_ catalyst demonstrated a higher benzene yield of 48% under the same conditions, indicating superior selectivity for aromatization.

As the reaction temperature increased, the formation of C₂–C₄ gaseous by-products increased gradually, reaching 9% at 450 °C, which resulted in decreased selectivity. This behavior can be related to the presence of acid sites on the γ-Al₂O₃ support, which enhance C–C bond hydrogenolysis at higher temperatures. However, the differences in selectivity between the US and MAS catalysts are more closely related to variations in metal–support interaction and platinum distribution, rather than acidity alone. The enhanced performance of the MAS catalyst can be attributed to stronger metal–support interactions and a more favorable balance between metallic and acidic functions, rather than simply higher platinum dispersion. As a result, the MAS-prepared catalyst demonstrates superior dehydrocyclization performance while reducing the formation of light gaseous by-products.

#### Ethanol conversion

**Fig. 13:**
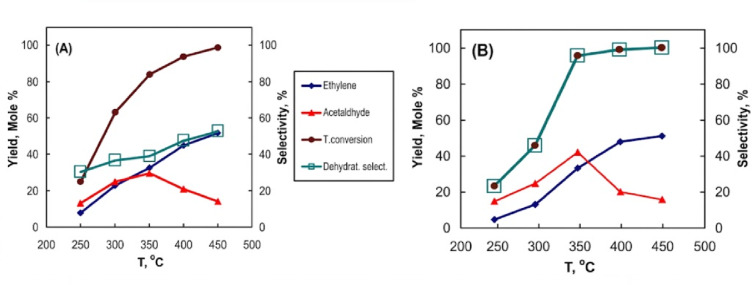
Catalytic conversion of ethanol over 0.9 wt% Pt/AC_2.5_ nanocatalysts using different methods: 0.9 wt% Pt/AC_2.5 (US)_ (A) and 0.9 wt% Pt/AC_2.5 (MAS)_ (B).

Figure 13 illustrates the catalytic conversion data of ethanol using Pt/AC_2.5_ nanocatalysts (0.9 wt% Pt) synthesized through ultrasound (US) and microwave-assisted synthesis (MAS) methods, within a temperature range of 250–450^°^C. All catalysts demonstrated significant activity for both the dehydration and dehydrogenation of ethanol. The dehydration of ethanol to ethylene over Pt/AC_2.5_ nanocatalysts begins at 250^°^C. As the reaction temperature increases, the activity parameters—total ethanol conversion, yield, and selectivity to ethylene improve Fig. 13**(A**,** B**). The catalysts 0.9 wt% Pt/AC_2.5 (MAS)_ and 0.9 wt% Pt/AC_2.5 (US)_ produced yields of 51% and 52% ethylene, respectively. The 0.9 wt% Pt/AC_2.5 (MAS)_ catalyst, synthesized via the microwave method, showed that ethanol dehydrogenation increases with temperature, reaching its peak at 350^°^C. At temperatures above 350 °C, the yield of acetaldehyde decreases because it participates in secondary reactions, such as decomposition and decarbonylation, which are expected to undergo secondary decomposition and decarbonylation reactions at high temperatures, as reported in the literature, resulting in light gaseous products such as CO and CH_4_. Furthermore, the dehydration pathway becomes more favorable at these higher temperatures, leading to increased ethylene production at the expense of acetaldehyde. At this temperature, the yield of acetaldehyde was 42% for the 0.9 wt% Pt/AC_2.5 (MAS)_ catalyst, compared to 33% for the 0.9 wt% Pt/AC_2.5 (US)_ catalyst. The catalytic results from various reactions show consistent trends, reinforcing the reliability of the experimental data. This behavior is closely linked to the enhanced Pt dispersion and optimized surface properties noted in the characterization results. Additionally, no diethyl ether was produced at lower temperatures using either catalyst method. Regarding the mechanism of ethanol dehydration, ethanol first adsorbs onto the acid sites of the catalyst surface, forming an oxonium ion (C_2_H_5_OH_2_^+^), which subsequently leads to the formation of ethylene through dehydration^73^. The moderate acid sites on the catalyst surface significantly influence the catalytic dehydration performance of ethanol, as illustrated in Eqs. **(10–11).** Ethanol hydrogenation primarily occurs on the platinum (Pt) metallic sites of the Pt/AC catalyst surface and involves several steps: adsorption, bond cleavage, and hydrogen evolution. Initially, ethanol molecules adsorb onto the metal surface via their oxygen atom, forming a surface ethoxy intermediate. This intermediate then dissociates the O–H bond, resulting in a surface-bound ethoxide species and an adsorbed hydrogen atom. Finally, the hydrogen from the ethoxide group is abstracted by the metal surface, resulting in the formation of acetaldehyde and an additional adsorbed hydrogen atom, as illustrated in Eq. 1**2**.

It is important to emphasize that the catalytic behavior of Pt/AC_2.5_ is driven by its bifunctional nature. The Pt sites serve as redox (metallic) centers that facilitate ethanol dehydrogenation through the activation of C–H and O–H bonds, leading to the production of acetaldehyde. In contrast, the acidic sites of the support are essential for the dehydration of ethanol to ethylene and for stabilizing reaction intermediates. Consequently, the overall catalytic performance and product distribution are significantly influenced by the interplay between the metallic (Pt) and acidic functions, which determines the selectivity for dehydrogenation versus dehydration pathways. All catalytic performance values reported in the text are fully consistent with the corresponding Table 7.

### Mechanism for dehydration of ethanol

11 $${\rm C_{2}H_{5}OH + M-O-M\rightarrow\:-H_{2}OM-O-M-O-CH_{2} -CH_{3} }$$

12 $${\rm CH_{3}-CH_{2} -O-M-O-M \rightarrow\:C_{2} H_{4} + M-O-M-OH}$$

### Mechanism for dehydrogenation of ethanol

13 $${\rm C_{2}H_{5}OH+ M-O- O-M CH_{3}CHO + M-O-M + H_{2} }$$

**Table 7:** Catalytic performance of 0.9 wt% Pt/AC_2.5_ catalysts in cyclohexane, n-hexane, and ethanol conversion at different temperatures.

Catalyst	Cyclohexane Conversion			*n*-hexane Conversion			Ethanol Conversion			
	Temp.(°C)	Benzene (%)	Total Conversion	Gases (C2–C4)	Benzene (%)	Total Conversion	Ethylene (%)	Water (%)	Acetaldehyde (%)	Total Conversion
**Pt/AC**_**2.5 (US)**_	450	75	75	9	40	**49**	52	33	14	**99**
	400	56	56	6	35	**41**	45	28	21	**94**
	350	48	48	0	30	**30**	33	21	30	**84**
	300	40	40	0	26	**26**	23	15	25	**63**
	250	–	–	0	22	**22**	8	4	13	**25**
**Pt/AC**_**2.5 (MAS)**_	450	86	86	–	48	**48**	51	33	16	**100**
	400	84	84	–	38	**38**	48	31	20	**99**
	350	57	57	–	35	**35**	33	21	42	**96**
	300	44	44	–	29	**29**	13	8	25	**46**
	250	–	–	–	23	**23**	5	3	15	**23**

## Study Limitations

This study has some limitations by the specific organic templates and reaction conditions employed for the in-situ reduction of platinum supported on γ- Al_2_O_3_. As a result, the catalytic performance observed may differ under alternative experimental setups or with other support materials. Furthermore, while this research emphasizes microwave and ultrasound-assisted reduction methods, it does not address other reduction techniques. To generalize the findings and gain a comprehensive understanding of the mechanisms involved, further investigations incorporating a wider variety of conditions, catalysts, and reduction methods are necessary.

## Conclusion

In this study, γ-Al₂O₃ nanopowders were successfully synthesized and modified using cetyltrimethylammonium bromide (CTAB) and Pluronic P123. The modified alumina sample, referred to as AC_2.5_, demonstrated enhanced surface and textural properties, making it an effective support for Pt-based nanocatalysts. The catalytic results indicate that the reduction method significantly affects catalyst performance. In cyclohexane dehydrogenation and n-hexane dehydrocyclization, the 0.9 wt% Pt/AC_2.5(MAS)_ catalyst exhibited higher catalytic activity than the ultrasonically prepared sample, highlighting the advantageous impact of microwave-assisted treatment on catalyst efficiency. In the conversion of ethanol, both MAS and US catalysts displayed similar performance in ethanol dehydration, achieving a maximum ethylene yield of approximately 52%. This suggests that the reduction method has a limited influence on this particular reaction pathway. The enhanced catalytic behavior of the MAS catalyst is attributed not only to improved Pt dispersion but also to stronger metal-support interactions and optimized surface properties. Although the US catalyst had slightly smaller Pt particle sizes, overall catalytic performance is influenced by a combination of structural and physicochemical factors rather than particle size alone. Overall, the findings underscore the significance of the synthesis and reduction methods in optimizing catalyst properties and establish a clear structure-activity relationship.

## Data Availability

The data supporting the findings of this study are included within the article.
